# Supplementary material for: Factor associated with anthropometric failure among under-five Bengali children: A comparative study between Bangladesh and India
Source: PLoS One. 2022 Aug 5;17(8):e0272634. doi: 10.1371/journal.pone.0272634 (PMC9355208; doi:10.1371/journal.pone.0272634)
Supplement: Attachment — Submitted filename: Response to Reviewers.docx [file pone.0272634.s001.docx]

**Response to Reviewers**	Date: March 11, 2022

**Paper Title:** Factor associated with anthropometric failure among under-five Bengali children: a comparative study between Bangladesh and India
**Journal Name:** PLOS ONE
**Paper ID:** PONE-D-21-41052

Dear Editor

Thank you very much for providing you and reviewers’ insightful remarks on our manuscript. We have made the necessary changes and revised the manuscript accordingly, and detailed point–by–point corrections are given below:

**Review Reports:**

1. Please provide additional details regarding participant consent. In the ethics statement in the Methods and online submission information, please ensure that you have specified (1) whether consent was informed and (2) what type you obtained (for instance, written or verbal, and if verbal, how it was documented and witnessed). If your study included minors, state whether you obtained consent from parents or guardians. If the need for consent was waived by the ethics committee, please include this information.

If you are reporting a retrospective study of medical records or archived samples, please ensure that you have discussed whether all data were fully anonymized before you accessed them and/or whether the IRB or ethics committee waived the requirement for informed consent. If patients provided informed written consent to have data from their medical records used in research, please include this information.

2. We note that you have indicated that data from this study are available upon request. PLOS only allows data to be available upon request if there are legal or ethical restrictions on sharing data publicly. For more information on unacceptable data access restrictions, please see http://journals.plos.org/plosone/s/data-availability#loc-unacceptable-data-access-restrictions.

Before we proceed with your manuscript, please address the following prompts:

a) If there are ethical or legal restrictions on sharing a de-identified data set, please explain them in detail (e.g., data contain potentially identifying or sensitive patient information, data are owned by a third-party organization, etc.) and who has imposed them (e.g., a Research Ethics Committee or Institutional Review Board, etc.). Please also provide contact information for a data access committee, ethics committee, or other institutional body to which data requests may be sent.

b) If there are no restrictions, please upload the minimal anonymized data set necessary to replicate your study findings to a stable, public repository and provide us with the relevant URLs, DOIs, or accession numbers. For a list of recommended repositories, please see

<https://journals.plos.org/plosone/s/recommended-repositories.> You also have the option of uploading the data as Supporting Information files, but we would recommend depositing data directly to a data repository if possible.

c) Please provide a link to the specific dataset on the Demographic and Health Surveys (DHS) website.

We will update your Data Availability statement on your behalf to reflect the information you provide.

**Reviewer 1**

The title is relevant and addresses the public health problem of under-five children. However, the following comments shall be considered in order to improve it. In addition, the entire manuscript requires extensive language editing.

**Response to Reviewer Comments:** Thank you very much for your comments/suggestions on our manuscript. We have tried with our best to revise our manuscript accordingly.

**Results**

**Reviewer Comment # 1:** Line 165-166 and under table 2, is there any statistically significant difference in the prevalence of anthropometric failure (AF) among under-five children in India and Bangladesh? If yes, please state it clearly?

**Response to Reviewer Comments:** Based on your point, we applied Z-proportional test to check if there was a significant difference in the categories of anthropometric failure (AF) between the under-five children of Bangladesh and India, and we noticed that there were significant differences [Line: 165-170; Page: 8-9].

**Reviewer Comment # 2:** On lines 201, 211 & 221, it is better to say “Factors associated with AF….” instead of saying “Predictors of AF…....”

**Response to Reviewer Comments:** Thank you for your suggestion. We have checked and made correction.

**Reviewer Comment # 3:** Lines 201-230, please incorporate the AOR with 95% CI at the end of each statement which deals with associated factors.

**Response to Reviewer Comments:** Thank you for your suggestion. We have kept the original content unaltered and added AOR with 95% CI to lines 201 to 230. In the case of variables with more than two categories have more than two AOR and 95% CI have to be mentioned, where we noticed that the ease of reading the sentences is reducing. In this situation, we have tried to mention AOR and CI as many as variables allows.

**Discussion**

**Reviewer Comment # 1:** Lines 233-240, shall be revised. It would be better to put the summary of the aim and method instead of stating it as such.

**Response to Reviewer Comments:** Thank you for your suggestion. We have added the summary of the aim and method at the beginning of the discussion and removed the sentences in lines 233-240, as you recommended.

**Reviewer Comment # 2:** For better communication and understanding, please state each factor in a paragraph instead of discussing more than one factor in a paragraph.

**Response to Reviewer Comments:** Thank you for your comment. We agree with you, we have described each factor in a separate paragraph. Which made the discussion a lot easier to read.

**Reviewer Comment # 3:** It is better to revise the strength and limitations of the study. If not, better to remove it since it does not seem the strength and limitations of the study.

**Response to Reviewer Comments:** Thank you for your suggestion. We have removed the point ‘strength and limitations of the study’ as per your suggestion.

**Conclusions**

**Reviewer Comment # 1:** Lines 297-299, the conclusion should be drawn based on the findings of the study. Thus, please remove the statement “Present study will contribute to achieving four SDGs of the 298 United Nations out of seventeen, no poverty, zero hunger, good health and well-being, and 299 quality education”. You have already stated it under the introduction section of the manuscript.

**Response to Reviewer Comments:** Thank you for your suggestion. The sentence you mentioned has been removed.

**Reviewer Comment # 2:** The conclusion, abbreviation, and acknowledgment should be written as a chapter heading on a separate line.

**Response to Reviewer Comments:** Thank you for your suggestion. We have checked and made correction.

**References**

**Reviewer Comment # 1:** Please cite the references properly. Don’t mix up it.

**Response to Reviewer Comments:** Thank you for pointing this out. In order to make some changes in the manuscript, the changes made in the reference case were revised and rearranged.

**Figure Legends**

**Reviewer Comment:** Lines 433-436, Figure captions should be self-explanatory. So, please revise it.

**Response to Reviewer Comments:** Thank you for your comment. We have modified figure’s caption.

**Reviewer 2**

**Reviewer Comment:** This manuscript is very good and well organized and constructed. It can be accepted and published, it will help the health professionals in understanding the compensate index of anthropometric failure.

**Response to Reviewer Comments:** Thank you very much for your valuable comment.

We would like to thank the reviewers for the valuable comments. We have revised the documents to the best of our ability, but we will definitely be happy to provide further improvement if there are further clarifications required.

With best regards

Dr. Md. Golam Hossain

Professor of Health Research Group

Department of Statistics, University of Rajshahi

Rajshahi-6205, Bangladesh

E-mail: hossain95@yahoo.com
