# Supplementary material for: Factor associated with anthropometric failure among under-five Bengali children: A comparative study between Bangladesh and India
Source: PLoS One. 2022 Aug 5;17(8):e0272634. doi: 10.1371/journal.pone.0272634 (PMC9355208; doi:10.1371/journal.pone.0272634)
Supplement: Attachment — Submitted filename: Response to Reviewers.docx [file pone.0272634.s002.docx]

**Response to Reviewers**	Date: June 26, 2022

**Paper Title:** Factor associated with anthropometric failure among under-five Bengali children: a comparative study between Bangladesh and India
**Journal Name:** PLOS ONE
**Paper ID:** PONE-D-21-41052R1

Dear Editor

Thank you very much for providing you and reviewers’ insightful remarks on our manuscript. We have made the necessary changes and revised the manuscript accordingly, and detailed point–by–point corrections are given below:

**Journal Requirements:**
Please review your reference list to ensure that it is complete and correct. If you have cited papers that have been retracted, please include the rationale for doing so in the manuscript text, or remove these references and replace them with relevant current references. Any changes to the reference list should be mentioned in the rebuttal letter that accompanies your revised manuscript. If you need to cite a retracted article, indicate the article’s retracted status in the References list and also include a citation and full reference for the retraction notice.

**Response for Journal Requirements:** We have checked the reference list and have found some mistakes that have been corrected.

**Review Reports:**

Reviewer #1: Dear authors,
You have addressed my comments partially. But, still, I have some concerns.
1. Lines 110-115: Ethical approval and consent to participate shall be stated next to the statistical analysis section.

**Response to Reviewer Comments:** Thank you very much for your comments/suggestions on our manuscript. According to your suggestion, we have stated “Ethical approval and consent to participate” next to the statistical analysis section [Line: 161-166].

2. Table and figure captions are not self-explanatory. Still, they need revision. So, please revise them again.

**Response to Reviewer Comments:** We have revised tables and figures captions.

3. In the result section on lines 218-219, 223, 226, 234-235, 240, 246-247; please interpret the AOR findings correctly. When the AOR is less than 1 it should be subtracted from 1 in order to interpret it.

**Response to Reviewer Comments:** We have interpreted AOR as per your suggestions.

4. Lines 336-349: The references should be cited properly. References 3-8 were structured in Harvard Style. Please structure them in Vancouver style instead of Harvard style.

**Response to Reviewer Comments:** Thank you very much for your comments. We have checked and made corrections.

5. Still the manuscript needs language editing.

**Response to Reviewer Comments:** We have tried with our best to make grammatical corrections.

We would like to thank the reviewers for the valuable comments. We have revised the documents to the best of our ability, but we will definitely be happy to provide further improvement if there are further clarifications required.

With best regards

Dr. Md. Golam Hossain

Professor of Health Research Group

Department of Statistics, University of Rajshahi

Rajshahi-6205, Bangladesh

E-mail: hossain95@yahoo.com
